# Optimization and Validation of FePro Cell Labeling Method

**DOI:** 10.1371/journal.pone.0005873

**Published:** 2009-06-11

**Authors:** Branislava Janic, Ali M. Rad, Elaine K. Jordan, A. S. M. Iskander, Md M. Ali, N. Ravi S. Varma, Joseph A. Frank, Ali S. Arbab

**Affiliations:** 1 Cellular and Molecular Imaging Laboratory, Department of Radiology, Henry Ford Hospital, Detroit, Michigan, United States of America; 2 Frank Laboratory, Radiology and Imaging Sciences Clinical Center, The National Institutes of Health, Bethesda, Maryland, United States of America; 3 Intramural Research Program, National Institute of Biomedical Imaging and Bioengineering, Bethesda, Maryland, United States of America; National Institute on Drug Abuse, National Institutes of Health, United States of America

## Abstract

Current method to magnetically label cells using ferumoxides (Fe)-protamine (Pro) sulfate (FePro) is based on generating FePro complexes in a serum free media that are then incubated overnight with cells for the efficient labeling. However, this labeling technique requires long (>12–16 hours) incubation time and uses relatively high dose of Pro (5–6 µg/ml) that makes large extracellular FePro complexes. These complexes can be difficult to clean with simple cell washes and may create low signal intensity on T2* weighted MRI that is not desirable. The purpose of this study was to revise the current labeling method by using low dose of Pro and adding Fe and Pro directly to the cells before generating any FePro complexes. Human tumor glioma (U251) and human monocytic leukemia cell (THP-1) lines were used as model systems for attached and suspension cell types, respectively and dose dependent (Fe 25 to 100 µg/ml and Pro 0.75 to 3 µg/ml) and time dependent (2 to 48 h) labeling experiments were performed. Labeling efficiency and cell viability of these cells were assessed. Prussian blue staining revealed that more than 95% of cells were labeled. Intracellular iron concentration in U251 cells reached ∼30–35 pg-iron/cell at 24 h when labeled with 100 µg/ml of Fe and 3 µg/ml of Pro. However, comparable labeling was observed after 4 h across the described FePro concentrations. Similarly, THP-1 cells achieved ∼10 pg-iron/cell at 48 h when labeled with 100 µg/ml of Fe and 3 µg/ml of Pro. Again, comparable labeling was observed after 4 h for the described FePro concentrations. FePro labeling did not significantly affect cell viability. There was almost no extracellular FePro complexes observed after simple cell washes. To validate and to determine the effectiveness of the revised technique, human T-cells, human hematopoietic stem cells (hHSC), human bone marrow stromal cells (hMSC) and mouse neuronal stem cells (mNSC C17.2) were labeled. Labeling for 4 hours using 100 µg/ml of Fe and 3 µg/ml of Pro resulted in very efficient labeling of these cells, without impairing their viability and functional capability. The new technique with short incubation time using 100 µg/ml of Fe and 3 µg/ml of Pro is effective in labeling cells for cellular MRI.

## Introduction

Current development of stem cell transplantation therapies and the optimization of transplantation parameters require efficient and non-invasive method for *in vivo* monitoring and trafficking of transplanted cells. *In vivo* cell tracking can be accomplished by using different imaging modalities, such as positron emission tomography (PET), single photon emission-computed tomography (SPECT) and magnetic resonance imaging (MRI). MRI is non-invasive, offering high spatial and temporal resolution and therefore is an excellent means for *in vivo* cell tracking. However, translation to routine clinical application of MRI cell tracking method is coupled to the availability of efficient cell labeling reagent that is not toxic and is incorporated in labeled cell in sufficient quantities for detection by MRI. Superparamagnetic iron oxide nanoparticles (SPION), such as ferumoxides, are a class of MRI contrast agents used for imaging pathology in the liver [1], and have also been used as an *ex vivo* cell labeling agent for various types of mammalian cells for *in vivo* cellular MRI. For example, SPIONs have successfully been used for the labeling of cancer cells [2], T cells [3], dendritic cells [4] and stem cells[5], [6] and the techniques used to labeled cells have been extensively studied [7], [8], [9], [10]. As previously reported, SPION labeling does not exhibit adverse effects on cell physiology [11], [12] and when combined with natural product cationic transfection reagents it is incorporated [8] intracellularly within endosomes [13]. Previously, we have reported a novel method for generating magnetically labeled cells that is based on combining ferumoxides and protamine sulfate (**FePro;** both FDA approved agents being used for off label indications) into SPION-transfection agent complex [6], [14]. Magnetically labeled cells can then be used as probes for localizing and monitoring physiological and/or pathological processes. However, the quantitative capacity of cellular MRI strongly depends on the ability to efficiently label cells with SPION and accurately quantify the intracellular incorporation of the iron nanoparticle. We previously reported [6], [14] a protocol based on initially generating **FePro** complexes in serum free media for the purpose of labeling and subsequently adding the complexes to the culture media and incubating cells overnight. However, for the efficient cell labeling this procedure required relatively prolonged incubation times and a relatively high dose of **Pro** (5–6 µg/ml) that generated large (inner radius of 200–500 nm) **FePro** complexes. These complexes were difficult to eliminate from the media and the external surface of the cells with simple cell washes and may create extra low signal intensity on MRI, when cells were injected locally. These low signal intensities on MRI from extracellular SPION complexes are not desirable in any studies when monitoring the migration of labeled cells by MRI.

The purpose of this study was to revise the current FePro labeling method and to determine if the use of a lower dose of **Pro** and the direct addition (without pre-mixing) of **Fe** and **Pro** to the cells is more efficacious than mixing the cells with pre-made FePro complexes that are generated in a separate labeling step. For this study, human glioma tumor cell line (U251) and human acute monocytic leukemia cell line (THP-1) were used to optimize the technique and served as model systems for attached and suspension cell types, respectively. These cells were labeled with three different ratio combinations of Ferumoxide (**Fe**) and Protamine Sulfate (**Pro**): (1) 100 µg/ml Fe and 3 µg/ml Pro, (2) 50 µg/ml Fe and 1.5 µg/ml Pro and (3) 25 µg/ml Fe and 0.75 µg/ml Pro. After 2, 4, 24 and 48 hours the labeling efficiency was determined by analyzing cell viability, intracellular iron concentration, and the MRI detection threshold of the labeled cells. The optimized labeling protocol was then used to label human T-cells, hHSC, hMSC and mNSC C17.2) to determine the effectiveness of the technique.

## Materials and Methods

### Ethics Statement

The use of human cord blood in this study was approved by a Henry Ford Health System Institutional Review Board (IRB). Written informed consent was obtained and the consent process was maintained under IRB-approved security protocol using an IRB-approved consent form and the process of consent.

### FePro labeling of cells

Human glioma cells (U-251, ATCC, Rockville, MD) were grown in T25 cell culture flask in Dulbecco's modified eagle's medium (DMEM) supplemented with 10% Fetal Bovine Serum (FBS) (Hyclone) until they reached 80% confluence. For the labeling purposes, media was aspirated and without disrupting the cellular monolayer cells were washed once with 1×PBS and covered with 2.5 ml of serum free RPMI 1640.

THP-1 cells (ATCC, Rockville, MD) were maintained in RPMI 1640 (Sigma, St. Louis, MO) supplemented with 10% FBS (Hyclone) and 0.05 mM 2-mercaptoethanol (Chemicon). For the purpose of labeling, THP-1 cells were suspended at the concentration of 4×10^6^ cell/ml in serum free RPMI and plated in 24 well plate cell culture dish, 0.5 ml per well.

Commercially available **Fe** (Feridex IV; Bayer-Schering Pharma, Wayne, NJ, USA) was first added to the cells by directly pipetting into the culture dish, immediately after which **Pro** (American Pharmaceuticals Partners, Shaumburg, IL, USA) was added in the same manner. **Pro** was supplied as 10 mg/ml of stock solution and was freshly diluted to a concentration of 1 mg/ml in distilled water at the time of use. Cells were labeled with the following concentration combinations of **FePro** complex: (1) 100 µg/ml Fe and 3 µg/ml Pro, (2) 50 µg/ml Fe and 1.5 µg/ml Pro and (3) 25 µg/ml Fe and 0.75 µg/ml Pro. After incubating the cells in the presence of FePro complexes for 15 minutes at 37°C, 5% CO2, complete growth media was added (2.5 ml per flask for U251 cells and 0.5 ml per well for THP-1 cells) and the labeling procedure was further continued for 2, 4, 24 and 48 h at 37°C, 5% CO_2_. Upon labeling, cells were harvested and washed two times with 1× PBS. Suspension type cells (THP-1) were collected by pipeting, while attached cells (U251) were harvested by trypsinization. After the last wash, FePro labeled cells were used for phantom preparation, viability and intracellular iron concentration assay, electron microscopy preparation and microscopic slides preparations for PB staining. A generalized flow chart for labeling cells is shown in **Table 1**.

**Table 1:** Basic procedure for FePro cell labelling.

step	Suspension cell types	Attached cell types
1	4×10^6^ cells/ml in serum free media	Cells at 80–90% confluence in serum free media
2	Add Fe and mix	Add Fe and mix
3	Add Pro and mix	Add Pro and mix
4	Incubate 15 minutes, 37°C, 5% CO_2_	Incubate 15 minutes, 37°C, 5% CO_2_
5	Add complete growth media to achieve 2×10^6^ cells/ml	Add complete growth media in the volume equal to the one of serum free media added at the first step of the procedure
6	Incubate for 4 h 37°C, 5% CO_2_	Incubate for 4 h 37°C, 5% CO_2_
7	Wash 3 times in 1×PBS	Wash 3 times in 1×PBS

### Trypan Blue viability assay

FePro labeled cells were suspended in 1× PBS at the concentration of 1×10^6^/ml and mixed with 0.4% of trypan blue dye at the 1∶1 ratio. Ten µl of this mixture was loaded into hemocytometer, after which cells were counted. Cells with an intact membrane excluded the dye and were considered as live cells. The percentage of live and dead cells was determined.

### Intracellular Iron Quantification

Quantification of intracellular iron FePro labeled cells was performed as previously described [14], [15]. Briefly, after labeling with FePro, cells were washed three times with 1× PBS to dispose of extracellular FePro. Triplicates of labeled and non-labeled cells (3×10^5^ cells per 1.5 ml microfuge tube) were centrifuged at 3000 rpm for 5 minutes. After discarding the supernatants, cell pellets were incubated at 110°C overnight (no cap on tubes). The next day, iron was dissolved by adding 1 mL of 5 M hydrochloric acid (HCl) to each tube and samples were further incubated at 60°C for 4 h. During this incubation step, tubes were capped to prevent acid evaporation. Then, 0.5 ml of acid solution from each tube was transferred to a separate 1.5 ml cuvette and absorbance was read at 351 nm. The average absorbance value for each sample was divided by the number of cells to determine the average iron concentration per cell. Iron concentration was determined using the standard curve obtained by plotting the known iron concentration of several dilutions of iron vs. absorbance.

### Staining for intracellular iron and determining the labeling efficiency

Aliquots of labeled and non-labeled control cells were transferred to glass slides and fixed with 4% paraformaldehyde. Prussian blue cellular staining was performed by incubating fixed cells in a mixture of 4% potassium ferrocyanide and 3.7% HCl (Perl reagent for PB staining) for 30 minutes. Slides were then washed and cells counterstained with nuclear fast red. For diaminobenzide (DAB) enhanced PB staining, the slides were reacted with activated 0.014% DAB, containing 0.03% hydrogen peroxide, for 10–15 minutes, washed 3 times with 1× PBS and then counterstained with nuclear fast red. The cells were evaluated by light microscopy and cells exhibiting blue intracellular particles were considered PB positive and the percentage of positive cells was determined within the four randomly selected fields of view.

### Magnetic Resonance Imaging

To determine the optimal time required for FePro to effectively label cells that can be detected by clinical MRI, phantoms containing labeled and unlabeled cells were made. After labeling, cells were washed 3 times in 1× PBS counted and placed into NMR tubes (tubes with no magnetic effects) at the concentration of 1×10^6^ cell in 1 ml of PBS. Cells were then mixed with 1 ml of 8% gelatin and quickly solidified in ice.

MRI of the cell gel phantoms was performed with a whole body 3 T (Signa Excite, GE Medical Systems, Milwaukee, WI) using dedicated small animal coils (Doty scientific, Columbus, NC). Two dimensional multi-echo T2*-weighted images were obtained to create R2* maps (1/sec) and the parameters used to acquire T2*-weighted images were: TR = 600 ms, TE = 10, 15, 20 and 30 using a 128×128 matrix, FOV = 40 mm, and NEX = 2. Effective slice thickness was 1 mm for T2*-weighted images and 13 slices were imaged for each tube. Based on voxel size there were estimated 49 cells per voxel.

### Image Processing and analysis

R2* maps were calculated from T2*-weighted images of the tubes. To generate the maps, the MR images were exported as DICOM images from 3 T to a personal computer. Image analysis was performed using Eigentool software that has a comprehensive set of functions for displaying, restoring, enhancing and analyzing images [16], [17]. T2* maps were calculated as the reciprocal of the negative slope of the logarithms of the T2*-weighted images and R2* maps were created by inverting T2* maps, respectively. Mean R2* values were measured in minimum of 5 different slices within a region of interest (ROI).

### FePro labeling of CD133+ hHSCs, hMSCs, mNSC C17.2 and CD3+ T cells and functional assessment of labeled T cells

To determine the effectiveness of the revised technique labeling procedure was performed by utilizing hMSCs and mNSCs, and hHSCs and sensitized T cells that underwent the same labeling procedure and analyses as described for attached (U251) and suspension type (THP-1) cells using 100 µg/ml of Fe and 3 µg/ml of Pro for 4 h. Human MSCs (Cambrex, USA) were grown in T25 cell culture flask in DMEM supplemented with 10% FBS and were labeled when they reached 80% confluence. Mouse NSC C17.2 (generated by Dr. Snyder's group and kindly provided by neurosurgery department at Cornell University) were grown in T25 cell culture flask in DMEM supplemented with 10% FBS and 5% Horse serum and were used for labeling when they reached 80% confluence. CD 133+ hHSCs and T-cells were isolated from human cord blood, with an IRB approved protocol, by Ficoll gradient centrifugation followed by immunomagnetic MACS® system (Miltenyi Biotec Inc, Auburn, CA) CD133+ and CD3+ selection, respectively. Isolated CD133+ HSCs were cultured as previously described [18]. In brief, cells were cultured in Stemline II medium (Sigma, St. Louis, MO, USA) containing 40 ng/ml stem cell factor, 40 ng/ml FLT3, and 10 ng/ml thrombopoietin (all from CellGenix, Antioch, IL, USA) for at least 5 days before labeling, with the cell concentration kept at 1×10^6^/ml. Isolated CD3^+^ cells were cultured in the presence of IL-2 (10 ng/ml) for 24 h, after which cells were co-cultured with γ-irradiated (35–40 Gy) tumor primed dendritic cells (10∶1 ratio). After 7 days of co-culture, T cells proliferated and were subjected to FePro labeling. After labeling, T cells were exposed to secondary stimulation by co-culturing with γ-irradiated tumor primed dendritic cells (10∶1 ratio). After 5 days of co-culture T cell proliferation was analyzed by performing MTT (3-[4,5-dimethylthiazol-2-yl] -2,5-diphenyl tetrazolium bromide) assay (Roche Molecular Biochemicals). The absorbance of the formazan product was measured at a wavelength of 570 nm with 750 nm (subtracted) as reference.

### Electron Microscopy

Electron microscopy analysis was performed on the cells that were fixed with 3% gluteraldehyde according to our previously reported protocol [13]. In brief, the collected cells were fixed with 3% gluteraldehyde, washed three times with 0.1 M sodium cacodylate buffer, re-suspended in 10% warm agarose in a microfuge tube and immediately centrifuged (at 6000–10 000 rpm) to collect the cells at the bottom. Once the agarose gel hardened, the tip of the microfuge tube was clipped off and the agar removed out the top of the tube. The area with the cells was cut out (usually at the tip of the agar) and then re-suspended in 0.1 M sodium cacodylate buffer until ready for processing. The cell-agar pellet was placed in 2% osmium tetroxide in 0.1 M Na cacodylate buffer for 2 h. The osmium was removed and cell-agar pellet was washed three times with 0.1 M Na cacodylate buffer. Cells were then fixed in 1% uranyl acetate in 0.1 N acetate buffer overnight and then fixed in a Lynx automated processor (EMS, Hatfield, PA, USA) with alcohol dehydration, propylene oxide dehydration and through epon infiltration. After hardening in an epon mold at 60°C overnight, the cell-agar pellets were sectioned at 50–70 nm and mounted on copper grids. The grids were stained in uranyl acetate or with uranyl acetate and lead citrate. The specimens were imaged with a transmission electron microscope.

### Statistical analysis

Each experiment was performed at least two times and each sample was tested in triplicate. Data are expressed as mean±SD. Statistically significant difference was determined with one way ANOVA analysis followed by Fisher's PLSD post-hoc test, when there were more than two groups. For analysis between two groups student-t test was used. A value of p<0.05 was considered significant.

## Results

### Labeling efficiency and intracellular iron concentration in FePro labeled THP-1 and U251 cells

**Figure 1:**
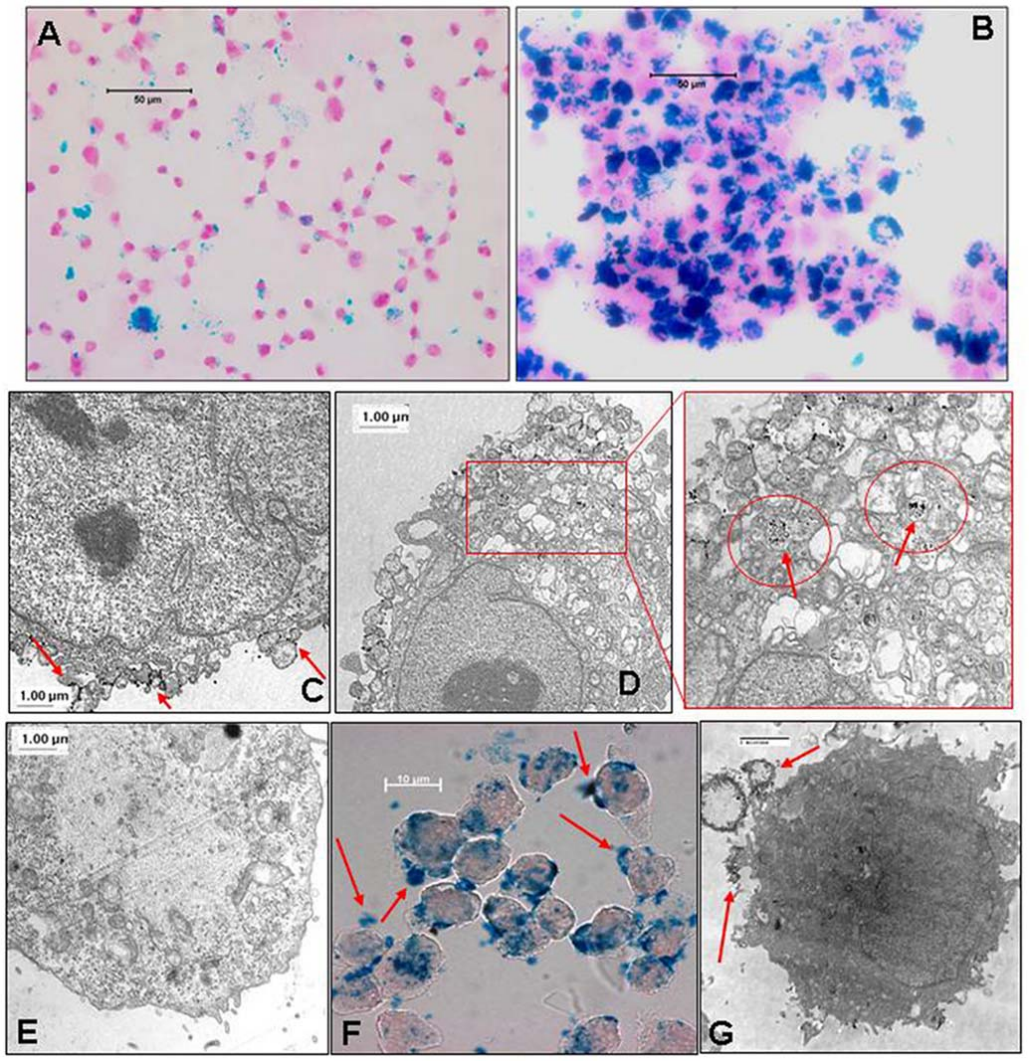
FePro labeled THP-1 and U-251 cells. Microphotography of cells labeled with 100 µg/ml Fe and 3 µg/ml Pro for 4 h and stained using PB method to visualize the intracellular iron incorporation in THP-1 cells (A, bar = 50 µm) and U-251 cells (B, bar = 50 µm). Electron microscopy images of U251 cells labeled with 100 µg/ml Fe and 3 µg/ml Pro for 4 h (C, D, bar = 1 µm) and control unlabeled U251 cells (E, bar = 1 µm). Note; extracellular FePro granules attached to the cell membranes and on their way to be engulfed and endocytosed (arrows) (C) and endocytosed intracellular iron within the endosomal compartment (arrows in circles) (D). Very little to no extracellular particles of FePro complexes seen compared to our previous method of labeling cells (F, G). Prussian blue staining of magnetically labeled hMSCs using our previous method showing extracellular complexes (F, bar = 10 µm) which are also confirmed by electron microscopic images (G bar = 2 µm). Extracellular complexes pointed by arrows in F–G.

**Figure 2:**
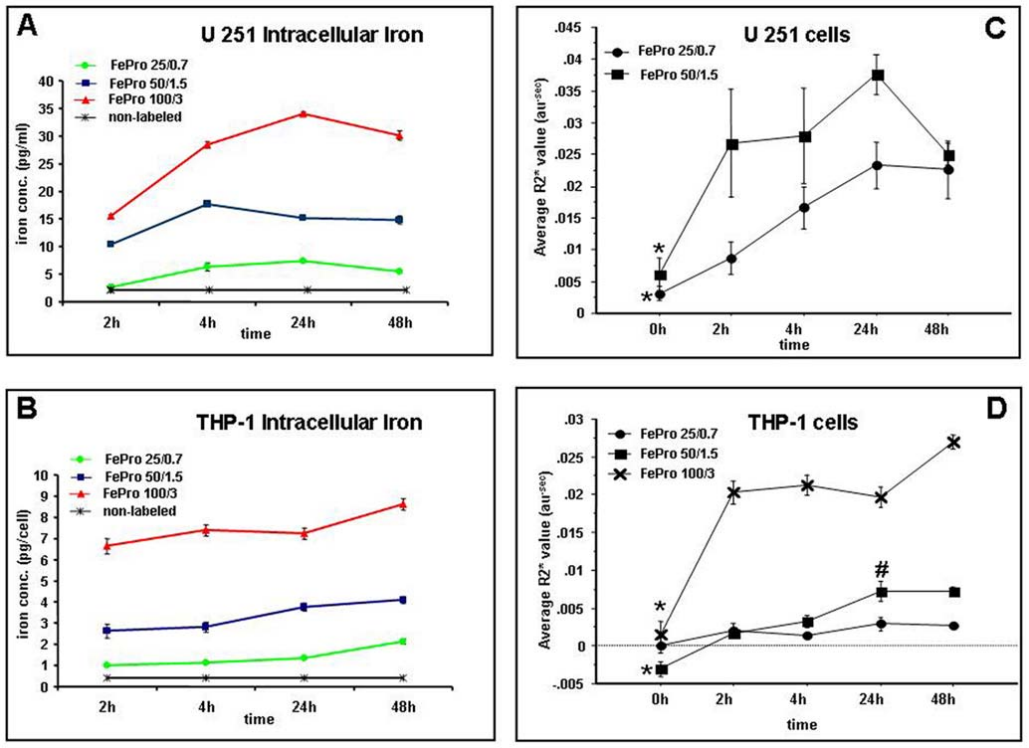
Intracellular iron concentration and MRI analyses of THP-1 and U251 phantom preparations. Iron concentration in U251 (A) and THP-1 (B) cells that were labeled with 100 µg/ml Fe and 3 µg/ml Pro (red), 50 µg/ml Fe and 1.5 µg/ml Pro (blue), and 25 µg/ml Fe and 0.75 µg/ml Pro (green). Cells were incubated for 2, 4, 24 and 48 h at 37°C, 5% CO2. Non-labeled cells (black). Data expressed as means±SD. *p<0.05 for cells labeled with 100 µg/ml Fe and 3 µg/ml Pro for 4 h vs. cells labeled with 0–50 µg/ml Fe and 0–1.5 µg/ml Pro. R2* values of U251 (C) and THP-1 (D) cells labeled with 100 µg/ml Fe and 3 µg/ml Pro (star), 50 µg/ml Fe and 1.5 µg/ml Pro (square), and 25 µg/ml Fe and 0.75 µg/ml Pro (circle). Data expressed as means±SD. *p<0.05 for unlabeled vs. labeled cells. ^#^p<0.05 THP-1 labeled with 50 µg/ml Fe and 1.5 µg/ml Pro for 4 h vs. THP-1 labeled with 50 µg/ml Fe and 1.5 µg/ml Pro for 24 h.

Qualitative analysis of FePro cellular uptake was performed on microscopic slide preparations of labeled cells that were stained with Prussian blue (PB). Representative bright light photomicrographs of THP-1 and U251 cells labeled with 100 µg/ml of Fe and 3 µg/ml of Pro for 4 h are shown in **Figure 1A and 1B**, respectively. Labeled cells exhibited abundant, heterogeneous uptake of FePro complexes that appeared as blue granules inside the cytoplasm on PB stained slides. Labeling efficiency analysis revealed that more than 95% of the cells were positive for PB. Electron microscopy (EM) analysis of U-251 cells labeled with 100 µg/ml of Fe and 3 µg/ml of Pro for 4 h, demonstrated the presence of fine FePro granules attached to the cell membranes and on their way to be macropinocytosed by the cells (**Figure 1C and 1D**), as well as intracellularly incorporated SPION within endosomes (**Figure 1D**). These data confirmed that 4 h time point was sufficient for generating intra-cytoplasmic, endosomal, iron oxide nanoparticle deposits (**Figure 1B–D** **)**. In addition, EM demonstrated a decrease in the amount of large FePro complexes that accumulated extracellularly when we used our previously published labeling technique [6], [14] (**Figure 1F–G**). After labeling with 100 µg/ml of Fe and 3 µg/ml of Pro, intracellular iron concentration in U251 cells reached the maximum of ∼35 pg-iron/cell at 24 h time point. The highest iron concentrations in U-251 cells labeled with 50 µg/ml of Fe and 1.5 µg/ml of Pro and 25 µg/ml of Fe and 0.75 µg/ml of Pro were observed between 4–24 h after labeling (**Figure 2A**). After labeling U251 cells for 4 hrs with 100 µg/ml of Fe and 3 µg/ml of Pro, these cells exhibited significantly higher intracellular Fe concentrations compared to non-labeled control cells or cells incubated with 25–50 µg/ml of Fe and 0.75–1.5 µg/ml of Pro (p<0.05). In THP-1 cells the highest intracellular Fe concentrations were observed at 48 h in cells incubated with 100 µg/ml of Fe and 3 µg/ml of Pro or with 25 µg/ml of Fe and 0.75 µg/ml of Pro. THP-1 cells incubated with 50 µg/ml of Fe and 1.5 µg/ml of Pro exhibited peak intracellular Fe levels at 24 h time point. However, incubating THP-1 cells for 4 h with 100 µg/ml of Fe and 3 µg/ml of Pro resulted in significantly higher intracellular Fe concentrations (p<0.05) compared to two other FePro concentrations used **(** **Figure 2B** **)**.

### Cellular Viability in FePro labeled U251 and THP-1 cells

The viability of FePro labeled U-251 and THP-1 cells was analyzed by trypan blue assay (**Table 2** **)**. We observed no significant differences in viability of the labeled U-251 and THP-1 cells at different time points and different labeling FePro concentrations, when compared to corresponding, non-labeled, controls.

**Table 2:** Percentage of viable THP-1 and U-251 cells at different time points after FePro labeling as assessed by Trypan blue viability test.

	25 Fe/0.7 Pro		50 Fe/1.5 Pro		100 Fe/3 Pro		Non-labeled	
	THP-1	U-251	THP-1	U-251	THP-1	U-251	THP-1	U-251
**2 h**	88.0%	83.3%	94.1%	86.1%	92.7%	86.6%		
**4 h**	90.0%	89.5%	94.7%	90.2%	92.0%	90.1%		
**24 h**	94.7%	82.8%	94.2%	84.3%	90.0%	89.0%	93.5%	88.7%
**48 h**	94.0%	82.5%	92.0%	86.3%	89.6%	85.6%		

### MRI results

Analysis of R2* values (1/sec) created from T2* images of the FePro labeled THP-1 and U-251 cell phantoms revealed incubation time and FePro concentration dependent changes (**Figure 2C–D**). Compared to unlabeled cell-phantoms, FePro labeled cells exhibited significant (p<0.05) increase in R2* values in all phantoms. For the FePro labeled U-251 cells this difference was observed even after 2 hours of incubation with the lowest concentration of Fe (25 µg/ml) and Pro (0.75 µg/ml) used. However, in THP-1 cells using the same labeling conditions (2 h incubation; 25 µg/ml of Fe and 0.75 µg/ml of Pro), the R2* values were not significantly increased compared to that of unlabeled cells. When compared among the incubation time, there was no significant difference in R2* values of cell phantoms between 4 and 24 hours of incubation within the respective concentration of Fe and Pro in both cell types (except THP-1 50/1.5 condition), indicating that 4 hours may be minimal and optimal time required for efficient cell labeling of these cell lines. In addition, the observed changes in R2* values corresponded to the results obtained by measuring iron concentration at indicated incubation time points **(** **Figure 2A–B** **)**.

### FePro labeling of CD3+ T cells and CD133+ hHSC

**Figure 3:**
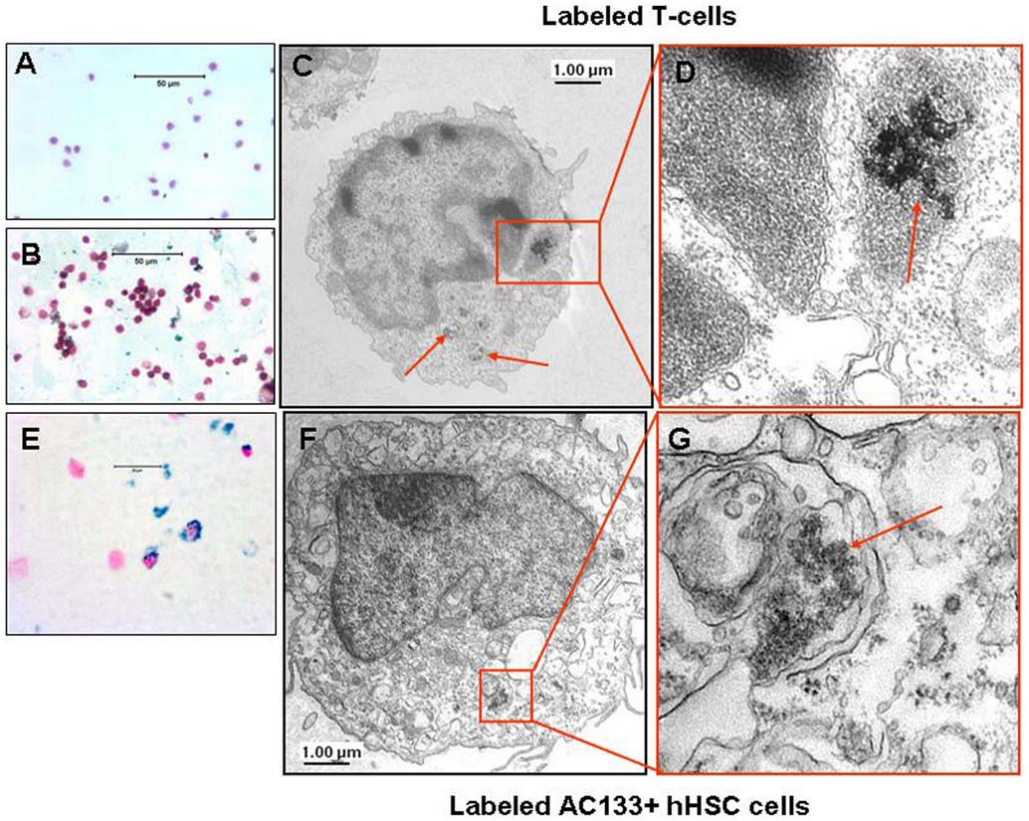
FePro labeled T and AC133+ hHSCs cells. Microphotography DAB-enhanced PB staining of non-labeled T-cells (A, bar = 50 µm) and T cells labeled with 100 µg/ml Fe and 3 µg/ml Pro for 4 h (B, bar = 50 µm). Note the intracellular localization of FePro complexes that appear as the dark brown granules on electron microscopic images (arrows). Electron microscopy images of T-cells (C–D, bar = 1 µm) and AC133+ HSCs (F–G, bar = 1 µm) cells labeled with 100 µg/ml Fe and 3 µg/ml Pro for 4 h. Prussian blue staining of AC133+ HSCs cells (E, bar = 20 µm).

To assess the effectiveness and validate the short incubation time-low FePro concentration revised technique in suspension type cells, activated CD3+ T-cells and CD 133+ hHSC were incubated with 100 µg/ml of Fe and 3 µg/ml of Pro for 4 h. T-cell immunological stimulation, manifested as proliferative T-cell response, was induced by co-culturing T-cells with γ-irradiated (35–40 Gy) tumor primed dendritic cells. After 7 days of co-culture, T-cells were incubated with FePro and analyzed for labeling efficiency, intracellular iron concentration and cell viability. As expected labeling efficiency, assessed by DAB-enhanced PB staining (**Figure 3A–B**) was greater than 95% and electron microscopy analysis showed intracytoplasmic/endosomal localization of internalized iron complexes (**Figure 3C–D**). In addition, intracellular iron concentration was significantly (p<0.05) higher compared to non-labeled, control T-cells (4.3 pg/cell vs. 0.19 pg/cell). Trypan blue analysis showed that the 4 hr incubation time labeling with FePro did not have any significant effect on T-cell viability (53%), compared to non-labeled control cells (55%). Most importantly, FePro labeled T-cells exhibited significant proliferative response after secondary immunological stimulation induced by 5 days co-culture with γ-irradiated tumor primed dendritic cells. This response, analyzed by MTT assay and expressed as optical density, exhibited no difference between labeled (0.279±0.0015) and control, non-labeled T-cells (0.260±0.003). These data indicate that the 4 hour incubation time and lower concentration of FePro in media did not alter T-cell functional status.

Incubating of CD133+ hHSCs with 100 µg/ml of Fe and 3 µg/ml of Pro for 4 h also resulted in efficient labeling with generating expected intracytoplasmic/endosomal iron deposits **(** **Figure 3E–G** **)**. Intracellular iron concentration was significantly (p<0.05) higher compared to non-labeled, control hHSCs (2.8 pg/cell vs.0.7 pg/cell) and had no significant effect on cell viability (p>0.05).

**Figure 4:**
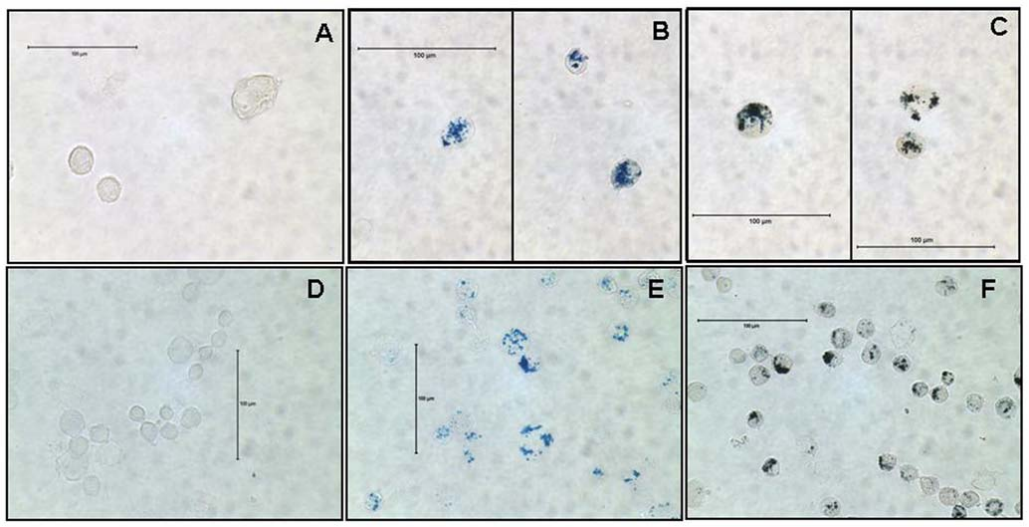
Unlabeled and FePro labeled hMSC (A–C) and mNSC (D–F) cells. Microphotography of PB staining of non-labeled (A) and PB (B) and DAB-enhanced PB (C) staining of hMSC cells labeled with 100 µg/ml Fe and 3 µg/ml Pro for 4 h (B–C, show images taken from two different fields in the same slide). PB staining of non-labeled (D) and PB (E) and DAB-enhanced PB (F) staining of mNSC cells labeled with 100 µg/ml Fe and 3 µg/ml Pro for 4 h. Note the intracellular localization of FePro complexes that appear as blue (PB stain) and the dark brown granules (DAB enhanced PB stain). (A–F, bar = 100 µm).

To validate the revised labeling technique in attached non-cancerous cell types, hMSCs and mNSCs C17.2 were incubated with 100 µg/ml of Fe and 3 µg/ml of Pro for 4 h. The presence of SPION in endosomes was confirmed with PB and DAB enhanced PB staining of hMSC **(** **Figure 4A–C** **)** and mNSCs **(** **Figure 4D–F** **)** cytospin preparations. In addition, analysis of intracellular iron concentration revealed the significant difference (p<0.05) between labeled and non-labeled hMSCs (19.1 pg/cell vs. 5.1 pg/cell) and between labeled and non-labeled mNSCs (10.1 pg/cell vs. 2.7 pg/cell) with no effect on cell viability (data not shown). It is possible that the higher basal levels of intracellular iron observed in non-label cells are due to the relatively high content of Ferric nitrate (0.1 mg/ml) in the complete media required for the growth of these cells.

## Discussion

The purpose of this study was to revise the current FePro labeling method [6], [14] based on initially generating **FePro** complexes in serum free media that are subsequently added to the culture media. Although this method usually generated excellent cellular *in vivo* MRI probes, it needed the improvements due to several drawbacks. For the efficient cell labeling this procedure required relatively prolonged (i.e., >12 hours) incubation times that may affect the cellular functional status. In addition, this method used a relatively high dose of **Pro** (5–6 µg/ml) that generated large **FePro** complexes that would attach to the outer cellular surface that could remain extracellular. These complexes were not always easy to eliminate from the media and the surface of the cells with simple cell washes and with heparin, and would often generate low signal intensity on MRI when cells were injected locally or are taken up by local tissue macrophages following direct implantation [19]. Hence, the low signal intensity generated as a result of extracellular FePro complexes may impede the accuracy of further quantitative MRI analysis.

In the present study, we have revised the current method and demonstrated that the optimal incubation protocol for the efficient labeling of adherent and suspension cells with FePro involve concentration ratio of 100 µg/ml of **Fe** and 3 µg/ml of **Pro** and the incubation time of 4 h at 37°C, 5% CO_2_. These conditions allow for the labeling of greater than 95% of cells in culture with no significant effect on cell viability. Although intracellular iron concentration in U251 labeled with 100 µg/3 µg FePro reached the peak at 24 h post labeling, elevated iron content levels were already achieved at 2 and 4 h of incubation. At the same time, the intracellular iron levels generated after incubating cells for 4 h with 100 µg/3 µg ratio of FePro were sufficient to create significant increase in R2* values on MRI analysis of labeled cells phantom preparations. In addition, MRI R2* values in phantom preparations of attached and/or suspension cell types were not significantly changed with increasing the concentration or cellular exposure time to FePro in culture media. Electron microscopy showed FePro located in endosomes without extracellular aggregates indicating that the achieved MRI detection threshold was due to the intracellularly incorporated ferumoxides. Therefore intracellular Fe levels obtained at 4 hrs were sufficient with regard to the MRI detection threshold in phantoms, eliminating the need for longer FePro labeling incubation time. Most importantly, validation experiments using T cells, CD133+ hHSCs, hMSCs and mNSCs revealed that incubating these cells for 4 h with 100 µg/ml of Fe and 3 µg/ml of Pro achieved high labeling efficiency without impairing the cellular viability. In addition, the functional properties of immunological T cell activity were not affected by internalized Fe complexes.

In conclusion, using the short incubation revised technique (**Table 1**) we achieved very efficient cell labeling, without impairing viability and functional capability that was successfully detected by MRI. Therefore, the new technique with short incubation time (4 h) using 100 µg/ml of Fe and 3 µg/ml of Pro that are directly added to the cells, is effective in labeling cells for cellular MRI. Importantly, this method holds enormous potential to be translated from bench to bedside using clinical grade plastics and materials in clinical good manufacturing practice facility. In addition, our new method can also be used for cell labeling with other commercially available SPIO nanoparticles that have similar zeta potential, dextran coating and sizes. Currently, few companies offer these similar SPIO nanoparticles available for research [20], [21], [22].
